# Correlation between PD-L1 expression and *MET* gene amplification in patients with advanced non-small cell lung cancer and no other actionable oncogenic driver

**DOI:** 10.18632/oncotarget.28045

**Published:** 2021-08-31

**Authors:** Marta Domènech, Ana M. Muñoz Marmol, Jose Luis Mate, Anna Estival, Teresa Moran, Marc Cucurull, Maria Saigi, Ainhoa Hernandez, Carolina Sanz, Alba Hernandez-Gallego, Aintzane Urbizu, Anna Martinez-Cardus, Adrià Bernat, Enric Carcereny

**Affiliations:** ^1^Medical Oncology Department, Catalan Institute of Oncology Badalona, Germans Trias i Pujol Hospital, Badalona, Barcelona, Spain; ^2^Pathology Department, Germans Trias i Pujol Hospital, Badalona, Barcelona, Spain; ^3^Badalona Applied Research Group in Oncology (BARGO), Institut d'Investigació en Ciències de la Salut Germans Trias i Pujol (IGTP), Badalona, Barcelona, Spain

**Keywords:** non-small cell lung cancer, oncogenic driver, PD-L1, *MET* amplification, smoking habit

## Abstract

Non-small cell lung cancers (NSCLC) are the most common type of lung cancer and can be classified according to the presence of mutually exclusive oncogenic drivers. The majority of NSCLC patients present a non-actionable oncogenic driver, and treatment resistance through the amplification of the *MET* *proto-oncogene* (*MET*) or the expression of programmed cell death protein 1 ligand (PD-L1) is common. Herein, we investigated the relation between *MET* gene amplification and PD-L1 expression in patients with advanced NSCLC and no other actionable oncogenic driver (i.e., *EGFR*, *ALK*, *ROS1*). Our retrospective observational study analyzed data from 48 patients (78% men, median age 66 years) admitted to the Germans Trias i Pujol Hospital, Spain, between July 2015 and February 2019. Patients presenting *MET* amplification showed a higher proportion of PD-L1 expression (93% vs. 39%; *p* < 0.001) and overexpression (64% vs. 27%; *p* = 0.020) than those with non-amplified *MET*. PD-L1 expression was not significantly different when analyzed by sex (*p* = 0.624), smoking history (*p* = 0.429), and Eastern Cooperative Oncology Group Performance Status (*p* = 0.597) Overall survival rates were not significantly affected by *MET* amplification (high and intermediate amplification vs low amplification and non-amplificated) (*p* = 0.252) nor PD-L1 expression (> vs =< 50%) (*p* = 0.893). In conclusion, a positive correlation was found between *MET* gene amplification and PD-L1 expression and highly expressed (above 50%) in patients with NSCLC and no other actionable oncogenic driver. It could be translated as new guided-treatment oportunities for these patients.

## INTRODUCTION

Cancer represents a heavy burden for society as a whole, with a high medical, economic, and psychosocial impact. Lung cancer is one of the most common cancers and has the highest death toll among them; 20% of all cancer-related deaths are attributed to lung cancer [1]. The vast majority of primary lung cancers are non-small cell lung cancer (NSCLC) [1]. NSCLCs can be classified at the molecular level according to the presence of oncogenic drivers that occur in genes crucial to tumor proliferation and survival. Several oncogenic drivers have been identified, which, in most cases, are mutually exclusive from one another [2]. However, only a fraction of them are druggable targets, also called actionable oncogenic drivers, for which targeted therapies are currently available. Among them, the most prevalent mutations in NSCLCs are seen in epidermal growth factor receptor (*EGFR*) (10–15%) followed by rearrangements in anaplastic lymphoma kinase (*ALK*) (3–7%) and *ROS1* (1–2%) genes [2, 3]. Nevertheless, most NSCLC patients present either a non-actionable oncogenic driver or an oncogenic alteration that has not yet been characterized [4, 5].

On the other hand, the emergence of treatment resistance is unavoidable [6]. In cases treated with EGFR-targeting tyrosine kinase inhibitors (TKI) (e.g., gefitinib, erlotinib), a common resistance mechanism occurs through the activation of the *MET proto-oncogene* (*MET*), also considered an oncogenic driver [6, 7]. In NSCLC, *MET* can either be activated through *MET* gene amplification, with a prevalence of 1–5%, or exon 14 skipping mutations, occurring in around 3% of NSCLCs [7]. The receptor tyrosine kinase encoded by *MET* is c-MET, whose ligand is the hepatocyte growth factor. Excessive c-MET activation in advanced cancers can cause tumor cell proliferation, motility, migration, and invasion [8]. Therapies with c-MET-TKIs (e.g., crizotinib, cabozantinib) have proven beneficial in lung cancer patients with *MET* gene amplification, preventing tumor growth, proliferation, and dissemination [9]. In addition, cancer cells can also achieve resistance through immune evasion. Programmed cell death protein 1 (PD-1) is expressed in T, B, and NK cells and, through the interaction with its ligand (PD-L1), allows the cells expressing it to evade the immune response through different mechanisms, among which exhaustion, apoptosis, and anergy [10]. In several cancers, including lung cancer, this immune checkpoint can be hijacked by inducing PD-L1 expression on tumor cells, which avoids the response of the host’s immune system [11, 12]. Therefore, immunotherapy with anti-PD-1 (e.g., nivolumab, pembrolizumab) and anti-PD-L1 (e.g., atezolizumab, avelumab) agents has yielded positive results in patients with advanced NSCLC [13].

Several studies have proven that PD-L1 expression is correlated with wild-type *EGFR* [12, 14, 15], *ROS1* rearrangement [14], and erlotinib-resistant NSCLC [6], while it is not associated with *ALK* mutations [16]. Besides, in some of these studies, *MET* gene amplification up-regulated PD-L1 expression, especially correlating with PD-L1 overexpression—considered as such for a tumor proportion score > 50% [6, 15]. However, to the best of our knowledge, no studies have been undertaken to explore the possible association of *MET* amplification and PD-L1 expression in advanced NSCLC patients presenting no other actionable oncogenic driver other than *MET.*

Therefore, the main aim of this study was to investigate the relation between *MET* gene amplification and PD-L1 expression in patients with advanced NSCLC and no other actionable oncogenic driver (i.e., *EGFR*, *ALK*, *ROS1*). We also analyzed the difference in PD-L1 expression according to sex, Eastern Cooperative Oncology Group Performance Status (PS), and smoking history of our studied population. Finally, we aimed to establish the effect of PD-L1 expression and *MET* gene amplification on the survival rates of our cohort.

## RESULTS

The baseline characteristics of our cohort are summarized in Table 1. Of the 50 patients eligible for our study, 39 (78%) were men, 43 (86%) were or had been smokers, and 74% had a PS of 0 or 1. The median age was 66 years (range: 44–82). Regarding histology, 38 (76%) patients presented an adenocarcinoma, 4 (8%) a squamous cell carcinoma, and on 8 (16%) the histology was not performed. In our cohort, 15 (30%) patients showed an intermediate or high *MET/CEP7* ratio, i.e., amplified *MET*. Positive PD-L1 expression was found in 27 (54%) patients, among which 19 (38%) showed high—over 50%—expression levels, i.e., PD-L1overexpression. In 2 (4%) patients PD-L1 was not possible to determine; hence, our analyses were performed on a total of 48 patients.

**Table 1:** Baseline characteristics of study participants

	Overall (*n* = 50)
**Sex**	
Male	39 (78)
Female	11 (22)
**Age (years), *median* (range)**	66 (44–82)
**Smoking history**	
Current	15 (30)
Former	28 (56)
Never	5 (10)
Not specified	2 (4)
**Histology**	
Adenocarcinoma	38 (76)
Squamous-cell carcinoma	4 (8)
Not specified	8 (16)
**ECOG performance status**	
0–1	37 (74)
2–3	13 (26)
**PD-L1 expression**	
Negative	21 (42)
1–50%	8 (16)
> 50%	19 (38)
Not determined	2 (4)
***MET* gene amplification **	
Non-amplified	35 (70)
Amplified	15 (30)

ECOG: Eastern Cooperative Oncology Group. Figures are absolute numbers (and %) unless otherwise stated.

**Figure 1:**
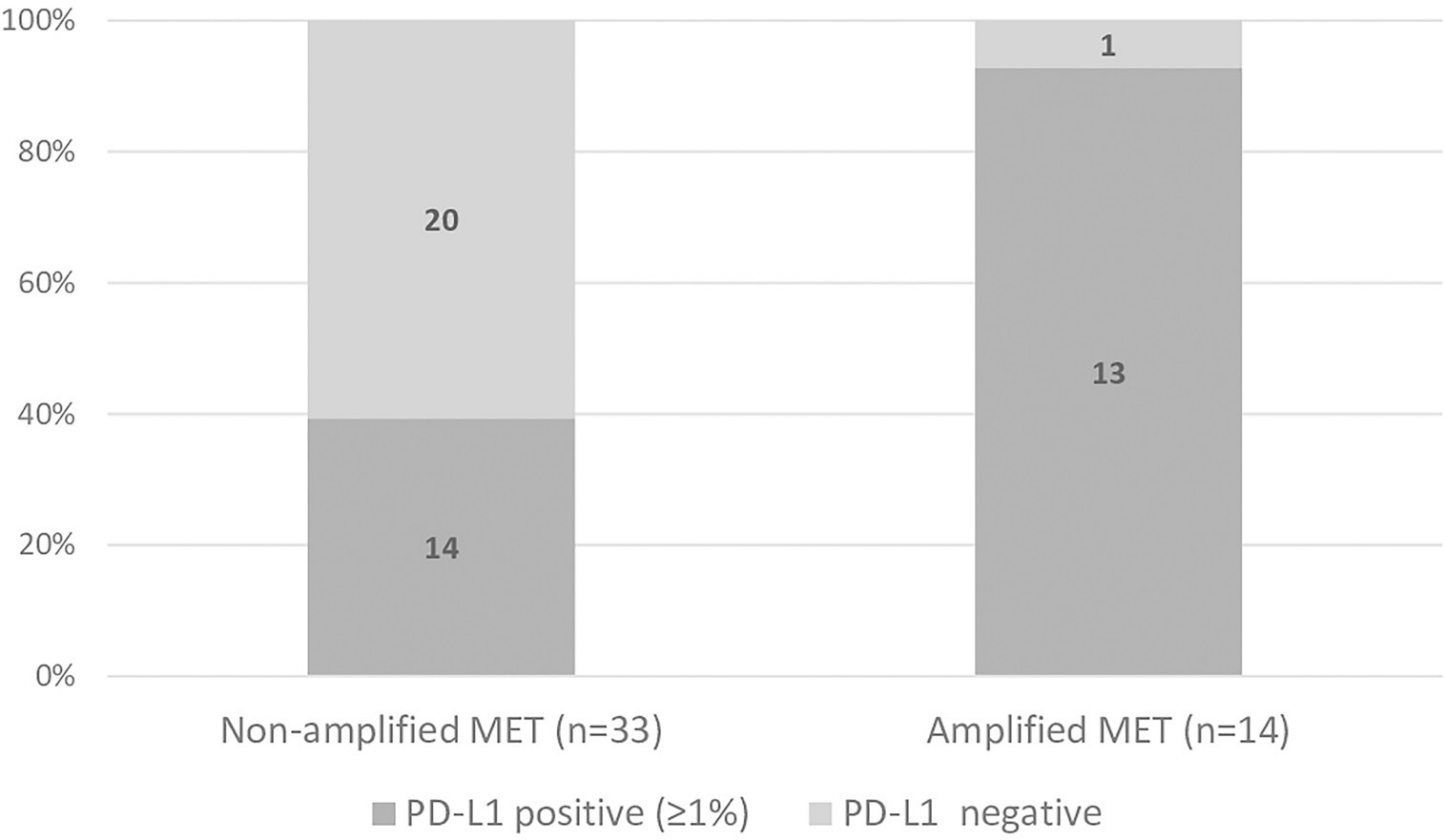
Proportion of patients presenting positive (≥ 1%) and negative PD-L1 in non-amplified and amplified *MET* groups. (Chi square test: *p* < 0.001).

**Figure 2:**
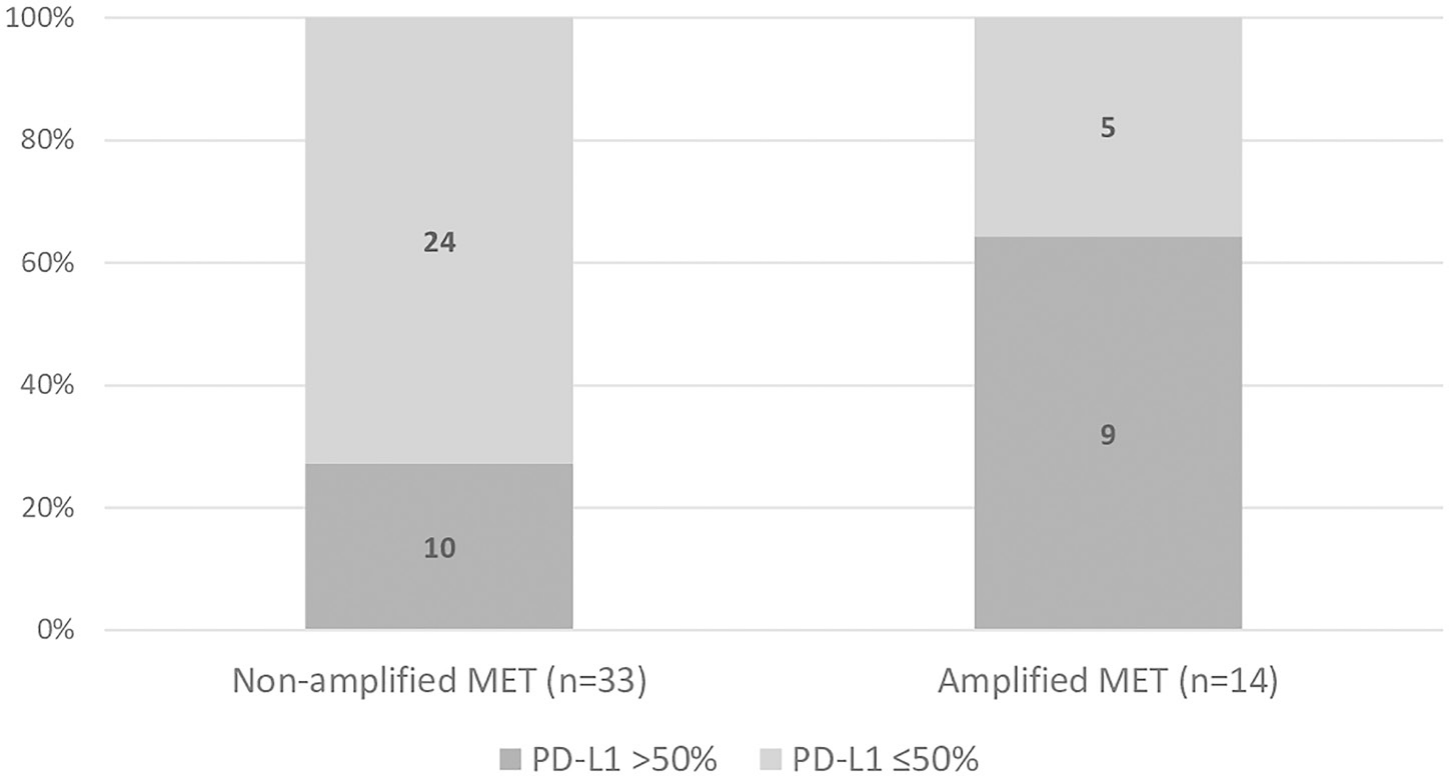
Proportion of patients overexpressing (> 50%) or not overexpressing (≤ 50%) PD-L1 in in non-amplified and amplified *MET* groups. (Chi square test: *p* = 0.020).

A higher proportion of positive PD-L1 expression was found among patients with amplified *MET* (93%) than among those with non-amplified *MET* (41%, *p* < 0.001) (Figure 1). Likewise, the proportion of patients overexpressing PD-L1 above 50% was higher among those with amplified *MET* (64%) than among those with non-amplified *MET* (27%, *p* = 0.020). However, most patients did not overexpress PD-L1 (*n* = 29, 62%) (Figure 2).

PD-L1 expression was not significantly different when analyzed by sex (*p* = 0.624), smoking history (*p* = 0.429), and PS (*p* = 0.597) (Table 2). However, the number of patients not overexpressing PD-L1 was invariably higher than those overexpressing it by sex, smoking history, and PS categories. Only in the subgroup of patients who had never smoked this trend was reversed, with a higher number of patients overexpressing PD-L1.

**Table 2:** PD-L1 expression stratified by sex, ECOG performance status, and smoking history

	PD-L1 ≤ 50%	PD-L1 > 50%	Overall (*n* = 48)	*p*-value
**Sex**				0.624
Male	23 (61)	15 (39)	38 (100)	
Female	6 (60)	4 (40)	10 (100)	
**Smoking history**				0.429
Current	8 (53)	7 (47)	15 (100)	
Former	17 (61)	9 (39)	28 (100)	
Never	2 (40)	3 (60)	5 (100)	
Not specified	2 (100)	0 (0)	2 (4)	
**ECOG performance status**				0.597
0–1	21 (60)	14 (40)	25 (100)	
2–3	8 (62)	5 (38)	13 (100)	

ECOG: Eastern Cooperative Oncology Group. Figures represent absolute numbers (and %).

**Figure 3:**
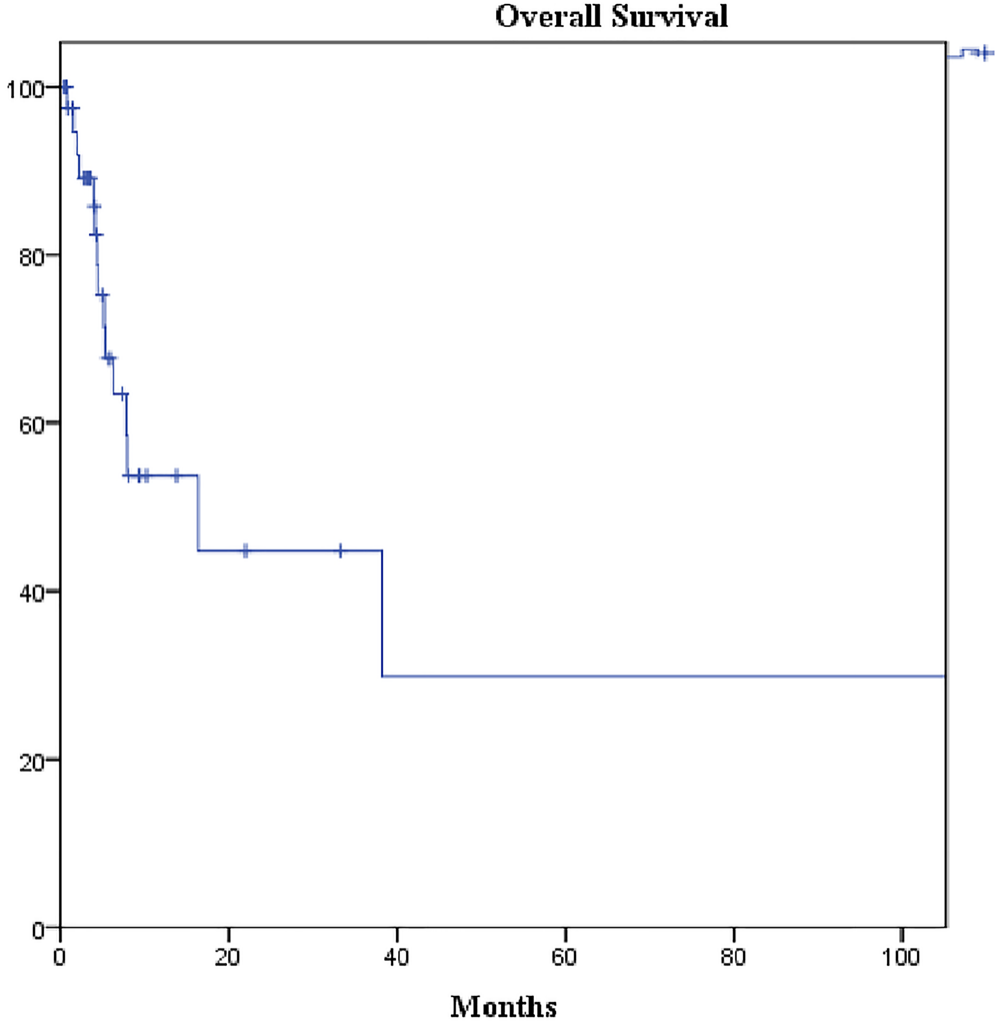
Kaplan-Meier curve of overall survival of the cohort. Median overall survival: 16.3 months (95% CI: 2.3–30.4).

**Figure 4:**
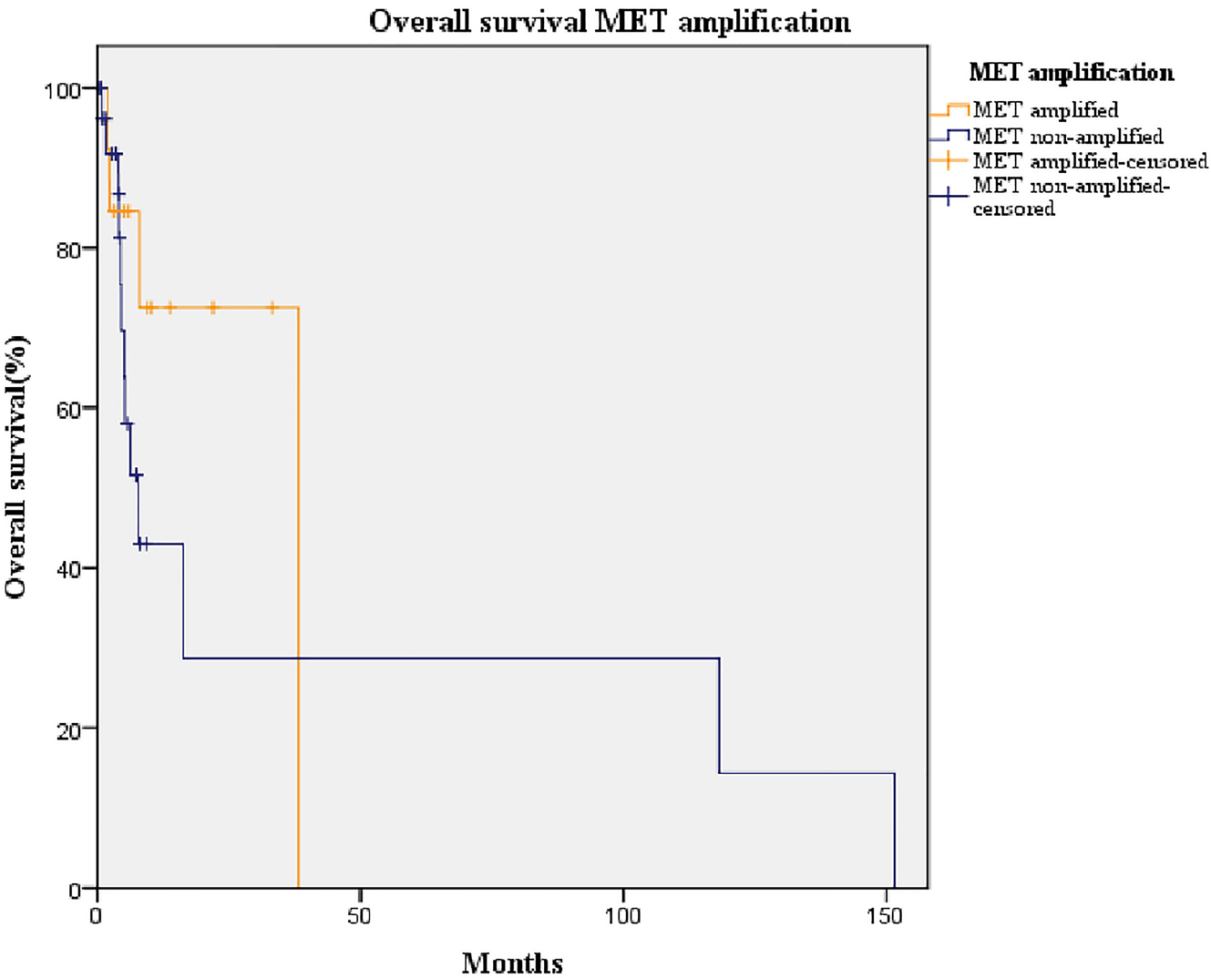
Kaplan-Meier curve of overall survival of patients with amplified *MET* (orange line) and non-amplified *MET* (blue line). Median overall survival of non-amplified *MET* group: 7.0 months (95% CI: 3.6–12.0). Median overall survival of amplified *MET* group: 38.0 months (95% CI: 5.7–70.5; *p* = 0.202).

**Figure 5:**
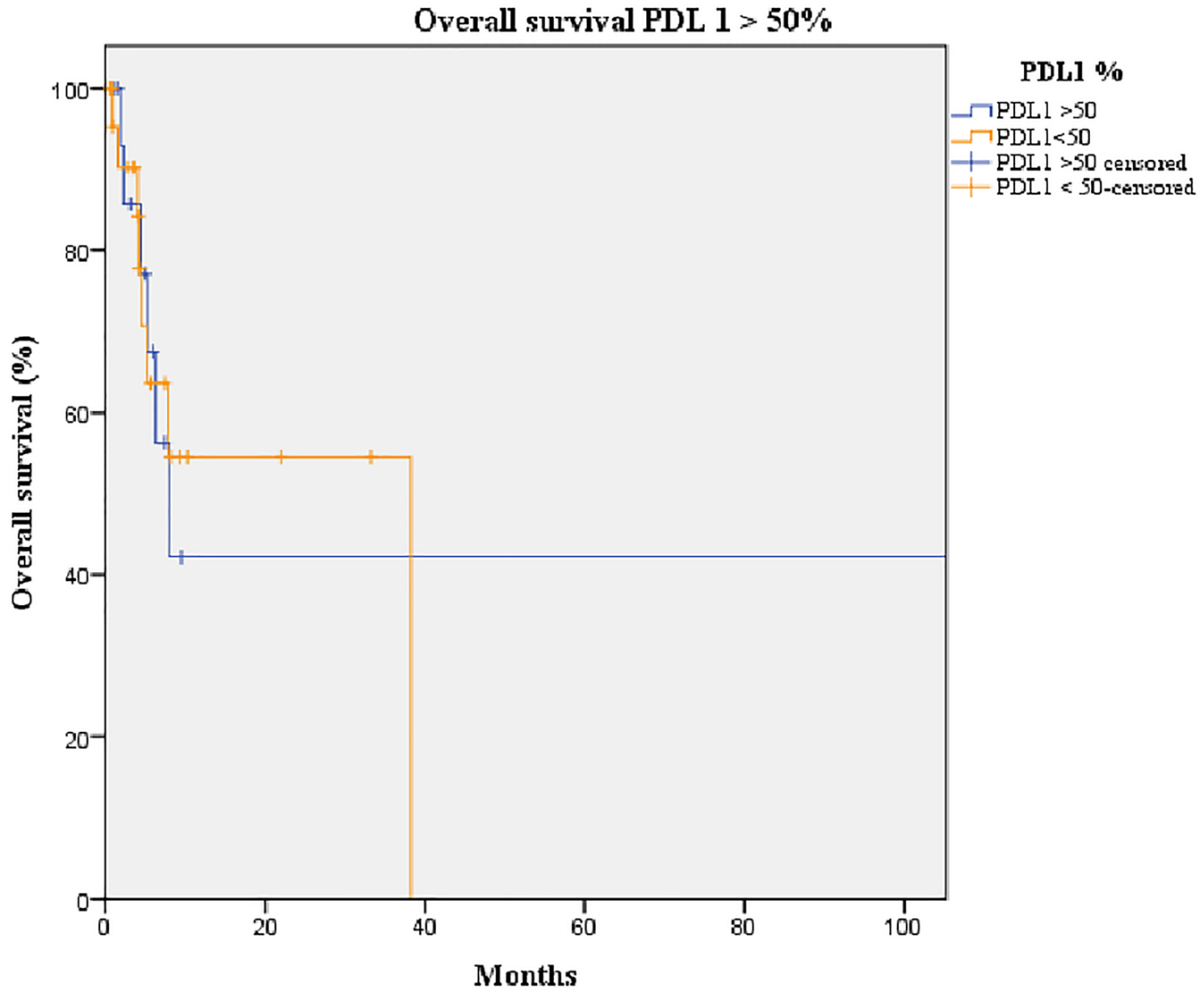
Kaplan-Meier curve of overall survival of patients overexpressing PD-L1 (orange line) and not overexpressing PD-L1 (blue line). Median overall survival of the group overexpressing PD-L1 (> 50%): 8 months (95% CI: 4.0–11.9). Median overall survival of the group not overexpressing PD-L1 (≤ 50%): 38 months (95% CI: 0–NR; *p*-value = 0.893).

The median follow-up duration was 30 months and the median overall survival (OS) of our cohort was 16.3 months (95% confidence interval [CI]: 2.3–30.4) (Figure 3). The difference in OS of patients with amplified *MET* (median: 38.2 months, 95% CI: 5.7–70.5) was not statistically significant from those with non-amplified *MET* (median: 7.8 months, 95% CI: 3.6–12.0; *p* = 0.252) (Figure 4). Likewise, no statistically significant difference in OS was found between patients overexpressing PD-L1 (median: 38.0 months, 95% CI: 0–93.8) and those not overexpressing it (median: 5.2 months, 95% CI: 0.7–9.6; *p*-value = 0.184) (Figure 5).

## DISCUSSION

To the best of our knowledge, this was the first study to show a positive correlation between *MET* gene amplification and PD-L1 expression in patients with NSCLC and no other actionable oncogenic driver (i.e., *EGFR*, *ALK*, *ROS1*). Indeed, although the absolute numbers were the same, the proportion of patients expressing and overexpressing PD-L1 was higher among those with amplified *MET*.

The baseline characteristics of our NSCLC cohort showed, as expected, a predominance of the male sex, current or former smokers, and adenocarcinoma. The ratios found in our study for positive PD-L1 expression (52%) and overexpression (36%) were in agreement with those reported previously on 791 NSCLC patients (63% of PD-L1 positive and 30% of overexpression) [17]. Likewise, a retrospective study on 389 NSCLC samples found 42% of positive PD-L1 expression and 19% of overexpression, also similar to our results [15]. Peculiarly, in our cohort, the number of patients showing positive PD-L1 expression or overexpression was the same in *MET* amplified and non-amplified groups, but these proportions were invariably higher in those exhibiting *MET* amplification. The correlation between *MET* amplification and PD-L1 expression has been previously studied in NSCLCs with other actionable oncogenic drivers, especially *EGFR*-mutation. The retrospective study abovementioned found that PD-L1 expression was correlated with *MET* amplification in proportions close to ours [15]. Also, in a small study performed on NSCLC patients with an EGFR-mutation, *MET* gene amplification was significantly associated with PD-L1 expression [18]. Besides, this correlation was demonstrated in an *in vitro* study with cells resistant to erlotinib [6]. Finally, our results are in agreement with those from a previous study that showed a higher occurrence of PD-L1 expression in *MET* amplified patients and demonstrated a positive correlation between *MET* amplification and PD-L1 expression [19].

Globally, more than 80% of lung cancers in men and almost 60% in women are caused by tobacco smoking [20]. Therefore, a correlation between smoking and PD-L1 expression is expected and has been repeatedly reported [21–23]. However, in our study, no correlation was found between PD-L1 expression and sex, smoking history, or PS. Interestingly, these results are in agreement with a large prospective study where none of these parameters had a significant impact on PD-L1 expression level [17] and with a previous meta-analysis in which only tumor differentiation showed a correlation with PD-L1 expression [10]. This could be explained, in our case, by the small size of our sample and the univariate analysis of data.

The OS of our cohort was in line with those reported for NSCLC patients with no actionable oncogenic driver [24]. However, *MET* amplification showed no statistically significant impact in OS, in contrast with previous studies [25–27]. This may be due to differences in the cut-off values used to define *MET* positivity or amplification, which lack consensus, and in the number of patients with stage IV tumors; in those patients, *MET* amplification did not further impact the OS [26]. However, in our study, the median OS of amplified (7.0 months) and non-amplified *MET* (38.0 months) groups displayed a non-negligible difference, but the small size of our sample precluded statistical confirmation of this result. Although PD-L1 has been found to be a poor prognosis factor [10, 16], we found that PD-L1 expression was not correlated with OS, as previously reported [28–30]. This could be explained, again, by the influence of the patients’ tumor stages and their ethnicity; no influence of PD-L1 expression has been reported on the OS of patients with stage IV tumors and was only an indicator of poor prognosis in Asian populations but not in non-Asians [16].

The main strength of our study was the homogeneity of the sample, which allowed, for the first time, to study the correlation between PD-L1 expression and *MET* gene amplification in patients with advanced NSCLC and no other actionable oncogenic driver. However, we were limited by the small sample size available and the inherent constraints of a retrospective study. In addition, the absence of a general consensus on cut-off values for *MET* amplification compelled us to use arbitrary ones. Therefore, we urge the community to arrive at this most needed consensus and to undertake prospective and large studies to validate or refute the results here described.

In conclusion, a positive correlation was found between *MET* gene amplification and PD-L1 expression and overexpression in patients with NSCLC and no other actionable oncogenic driver. In our study, PD-L1 expression was not affected by sex, PS, or smoking history, and PD-L1 expression and *MET* gene amplification did not affect the OS rates of our cohort. Further studies are needed to appraise the impact this finding may have on possible treatments for these patients.

## MATERIALS AND METHODS

### Study design

This was a retrospective observational study carried with data recorded in the Spanish Thoracic Tumor Registry (TTR), a National Registry of lung cancer cases managed and sponsored by the Spanish Lung Cancer Group (SLCG) (*Grupo Español de Cáncer de Pulmón*). The SLCG is an independent cooperative group formed mostly by oncologists and counting more than 500 members. In 2015, the SLCG decided to start a nationwide multicenter epidemiological study aimed at ascertaining the characteristics of lung cancer cases, their treatments, and survival, in an effort to offset the existing lack of information caused by the absence of a cancer registry with national coverage. Current regional registries barely cover 30% of the Spanish population and only exist in some particular regions. The TTR was opened to all Spanish hospitals and the first patient was enrolled in August 2016. The recruitment is still ongoing with more than 75 hospitals taking part. The methodology group of the SLCG designed specifically an electronic questionnaire to be used by the TTR. The information from all participants was entered through an electronic questionnaire by clinicians. The questionnaire had different sections: 1) demographic data (gender, age, etc.); 2) detailed history of tobacco use; 3) lung cancer characteristics at diagnosis (including a full list of possible symptoms); 4) all treatments received (with detailed information on each); 5) presence of specific mutations in driver genes at diagnosis; 6) disease progression; and 7) survival data.

### Patients

This study used data from patients admitted to the Catalan Institute of Oncology Badalona Germans Trias i Pujol Hospital between July 2015 and February 2019. We collected data recorded in the Spanish TTR from patients of our center. We included data from patients with advanced NSCLC and whose *MET* expression had been analyzed and showed no other mutation on relevant genes (i.e., *EGFR*, *ALK*, *ROS1*).

The TTR has been approved by the Clinical Research Ethics Committee of Puerta de Hierro University Hospital.

### Genotyping

We evaluated *MET* amplification by fluorescence *in situ* hybridization (FISH) using the Zyto Light^®^ SPEC MET/CEN 7 Dual Color Probe. Briefly, we deparaffinized 4-μm-thick paraffin-embedded tissue sections and processed them with the Histology FISH Accessory Kit (Dako). After pretreatment and enzymatic digestion according to the manufacturer’s instructions, we codenatured slides for 4 min at 85°C and hybridized them overnight at 37°C on a Hybridizer (Dako). Following hybridization, we removed coverslips and washed slides at 65°C for 2 min in 2 X SSC/0.3% Tween-20. After dehydration in a graded ethanol series, we counterstained samples with fluorescence mounting medium containing 4′,6-diamidino-2-phenylindole (DAPI). Finally, we scored hybridization signals in at least 20 non-overlapping nuclei.

No consensus exists on the most appropriate diagnostic cut-off point for *MET* amplification. Several approaches based either on gene copy number, the ratio of *MET* to chromosome enumerating probe against chromosome 7 (*CEP7*), or a combination thereof have been proposed [31]. In our study, we applied the criteria of Noonan et al. [32], who categorized samples in four groups conforming to *MET/*CEP7 ratios: not positive (< 1.8), low (≥ 1.8 to ≤ 2.2), intermediate (> 2.2 to < 5) and high amplified (≥ 5). Accordingly, we classified our samples with *MET/CEP7* ratio ≤ 2.2 (not positive or low) as non-amplified *MET* and those with *MET/CEP7* ratio > 2.2 (intermediate or high) as amplified *MET*.

### Immunohistochemistry

We processed tissue slides in a BenchMark ULTRA platform instrument (Ventana Medical Systems, Roche, Tucson, AZ, USA) and stained them with SP263 antibody, which we prediluted according to the manufacturer’s instructions. Following the established recommendations, we quantified the percentage of cells with membrane positivity (partial or complete expression) for each tumor. We did not assess tumor necrosis areas and discarded cases in which at least 50 viable cells were not available [33]. We determined the tumor proportion score by calculating the percentage of tumor cells membrane staining at any intensity. We considered PD-L1 expression in tumor cells positive if ≥ 1% of tumor cells had membranous staining of any intensity and high if > 50%.

### Statistical analysis

We described categorical variables as absolute frequencies and percentages and continuous variables as median and ranges. We compared categorical variables using the chi-square test. We calculated OS from the date of diagnosis to the date of last follow-up or death. We conducted survival analysis with the Kaplan-Meier method and we compared differences among groups and subgroups with the log-rank test. We set the statistical significance level at *p* < 0.05.
